# Evaluation of Inhibitory Action of Novel Non β-Lactam Inhibitor against *Klebsiella pneumoniae* Carbapenemase (KPC-2)

**DOI:** 10.1371/journal.pone.0108246

**Published:** 2014-09-29

**Authors:** Arbab Khan, Mohammad Faheem, Mohd Danishuddin, Asad U. Khan

**Affiliations:** Medical Microbiology and Molecular Biology Lab, Interdisciplinary Biotechnology Unit, Aligarh Muslim University, Aligarh, India; University of Cambridge, United Kingdom

## Abstract

The use of three classical β-lactamase inhibitors (Clavulanic acid, tazobactam and sulbactam) in combination with β-lactam antibiotics is presently the mainstay of antibiotic therapy against Gram-negative bacterial infections. However these inhibitors are unable to inhibit carbapenemase KPC-2 effectively. They being β-lactam derivatives behave as substrates for this enzyme instead of inactivating it. We have initiated our study to check the *in vitro* inhibition activity of the two novel screened inhibitors (ZINC01807204 and ZINC02318494) in combination with carbapenems against KPC-2 expressing bacterial strain and their effect on purified enzyme KPC-2. The MIC values of meropenem and ertapenem showed maximum reduction (8 folds) in combination with screened compounds (ZINC01807204 and ZINC02318494). CLSM images also depicted their strong antibacterial activity in comparison to conventional β-lactamase inhibitors. Moreover no toxic effect has been shown on HeLa cell line. Though the IC_50_ value of ZINC01807204 was high (200 µM), it exhibited fairly good affinity for KPC-2 (K_i_ = 43.82 µM). With promising results this study identifies ZINC01807204 as a lead molecule for further optimization and development of more potent non β-lactam inhibitors against KPC-2.

## Introduction

**Figure 1:**
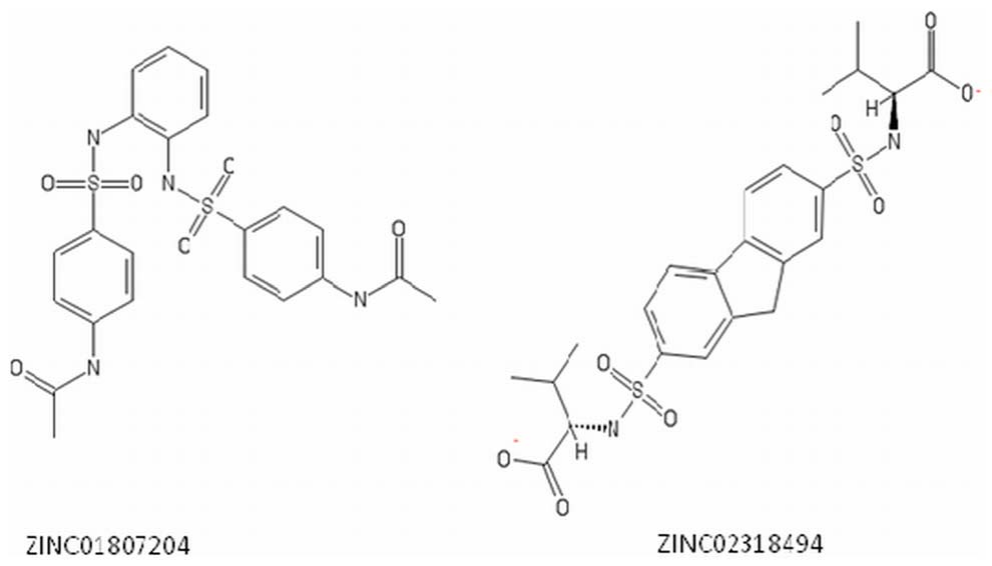
Chemical structure of screened inhibitors (A) ZINC01807204 (B) ZIC02318494.

*Klebsiella pneumoniae* carbapenemase (KPC) is a molecular class A serine β-lactamase belonging to functional group 2f [1]. First identified in *K. pneumoniae* clinical isolate from North Carolina in 2001, KPC has since been detected in many other Gram negative bacteria worldwide [2], [3]. KPC type β-lactamase exhibits broadest substrate profile conferring resistance to virtually all β-lactam antibiotics including carbapenems, severely restricting treatment options in patients infected with bacteria producing KPC and making it a serious threat both in community and nosocomial settings [4]. KPC have drawn the attention of microbiologists worldwide because of its unique structural characteristics. It has an overall structure like class A serine β-lactamases but share very little sequence similarity with other class A β-lactamases like CTX-M-1, TEM-1 and SHV-1 [5]. It possesses a large and shallow active site which can accommodate bulkier β-lactams easily making KPC-2 being dubbed as a “versatile β-lacatamse” [6], [7]. KPC are not only good in hydrolysing carbapenems, recent reports suggest their resistance against inhibitors as well. Commercial β-lactamase inhibitors like clavulanic acid, tazobactam or sulbactam are unable to inhibit this enzyme effectively [5], [8]. The activity of β-lactam antibiotics and β-lactamase inhibitors in combination have been successful in treatment of various infections caused by resistant bacteria but the KPC-2 β-lactamase have evolved to hydrolyze β-lactam inhibitors as substrates [9]. Thus search for new and efficient inhibitors is necessary to restore the antibacterial activity of current antibiotics. Keeping in view the above background, a study was initiated in our lab to find out novel non β-lactam inhibitors by structure-based virtual screening of ZINC database. The study concluded two potential inhibitors (ZINC01807204 and ZINC02318494) as effective inhibitor candidates against KPC-2 producing bacterial strains (Figure 1). In the present study, we went further to test the antibacterial efficacy of various carbapenems/ZINC01807204/ZINC02318494 combinations in vitro on bacterial cells. We investigated and compared the inhibition potency of screened inhibitors on purified KPC-2 enzyme with that of conventional inhibitors. We also studied the toxicity effect of these compounds and further propose these novel inhibitors as potential scaffolds for further optimization against KPC-2.

## Materials and Methods

### Ethic statement

A formal consent from the institutional ethical committee was taken and clearance was obtained from the institute’s ethics committee. Participants, and guardians, provided written, informed consent to participate in the study. Only single strain was obtained for this study. We have a specific format to get the consents of patients/parents of minors. These formats are according to the Institutional ethics committee’s guidelines. These forms are confidential and cannot be disclosed as per the guide lines. Institutional ethical committee has already approved. The name of committee/board is “The Bio-Ethical Committee at J.N. Medical College & Hospital” at AMU, Aligarh. The committee also decided that the study was exempted from full review.

### Acquisition of bacterial strain and its characterization

A carbapenem resistant clinical strain of *K. pneumoniae* (NP6), previously characterised in our lab was procured from Jawaharlal Nehru Medical College (JNMC), Aligarh [10]. The strain was found to harbour KPC-2 resistance marker. *Escherichia coli* (DH5α) and *E. coli* BL21 (DE3) were used for cloning and protein expression experiments respectively. Minimum inhibitory concentration (MIC) and Confocal Laser Scanning Microscopy (CLSM) experiments were conducted on *E.coli* (BL21) cells expressing recombinant KPC-2 β-lactamase.

### Cloning of bla_KPC-2_

The *bla*_KPC-2_ determinant was amplified from the parent strain, *K. pneumoniae* NP6, by using the protocol and primers for *bla*_KPC-2_ described previously [10]. Briefly, the plasmid DNA harboring the gene *bla*_KPC-2_ from clinical strain of *K. pneumoniae* (NP6) was extracted and amplified by PCR using primers KPC-F (5′ ATATCATATGTCACTGTATCGCCGT 3′) containing *Nde I* restriction site and KPC-R (5’ ATATCTGCAGTTACTGCCCGTTGACGC3’) having restriction site for *Pst I*. The cloning vector, pQE-2, and the PCR product were double digested by *Nde I* and *Pst I*, ligated and subsequently transformed into competent *E.coli* BL21 (DE3) cells by heat shock method. Transformed colonies were selected on ampicillin (100 µg/ml) containing agar plate. Clones were confirmed by colony PCR and restriction digestion method.

### Expression and Purification of Recombinant KPC-2

To express and purify KPC-2 carbapenemase, the pQE-2 vector harboring *bla*_KPC-2_ gene was transformed into competent *E. coli* BL21 (DE3) cells. These transformants were grown in Luria Bertani (LB) broth containing 100 µg/ml ampicillin at 37^ο^C with shaking till their optical density at 600 nm reached 0.6. Expression of recombinant β-lactamase gene was induced by adding 0.2 mM IPTG (isopropyl-D-thiogalactopyranoside) and the cells were again allowed to grow at 20^ο^C over night. Bacteria were harvested by centrifugation at 8000 rpm (Sigma rotor 12150H) for 10 minutes. The pelleted cells were resuspended in lysis buffer containing 50 mM sodium dihydrogen orthophosphate (pH 8) and 150 mM sodium chloride. The cells were ruptured by sonication using Vibra-cell sonifier (Sonar, 500 W) on ice at 25 pulse, altering between 9 s with 30% amplitude. Cell debris were removed by centrifugation at 12000 rpm for 30 minutes. Clear cell lysate was loaded on Ni-NTA column pre-equilibrated by lysis buffer. Washing of the column was done by lysis buffer supplemented with 20 mM imidazole. Recombinant protein was eluted using 300 mM imidazole. Pure protein was obtained after dialysis of eluted fractions against 50 mM phosphate buffer pH 8.0 containing 100 mM sodium chloride. Purity of the purified protein was assessed by sodium dodecyl sulphate polyacrylamide (SDS) gel electrophoresis. Gels were stained with Coomassie brilliant blue R250 (Figure S1). Protein concentrations were determined by measuring absorbance at λ = 280 nm and using the protein’s extinction coefficient (Δε; 39,545 M^−1^cm^−1^ at 280 nm) [11].

### Antibiotic Susceptibility Tests

The Minimum Inhibitory Concentrations (MIC) of recombinant BL21(DE3) cells harboring *bla*_KPC-2_ gene were determined by micro dilution method for different carbapenems alone and in combination with inhibitors (Clavulanic acid, Sulbactam, Tazobactam, ZINC01807204 and ZINC02318494) against BL21 control cells (cells with null vector). The recombinant *E. coli* cells were induced with IPTG prior to performing experiments. The antimicrobial agents, imipenem, meropenem, ertapenem, clavulanic acid, sulbactam and tazobactam were obtained from Sigma Aldrich (USA) and compounds ZINC01807204 and ZINC02318494 were procured from Vitas M (Netherlands). The inhibitors were taken at a fixed concentration of 4 µg/ml. The results were interpreted according to the guidelines laid by Clinical Laboratory Standards Institute (CLSI) [12]. The MIC was determined as the lowest concentration that totally inhibits visible bacterial growth.

### Confocal Laser Scanning Microscopy (CLSM) for Live/Dead cell staining

To analyse the effect of novel inhibitors, cloned *E.coli* cells (BL21) harbouring KPC-2 marker were induced and grown in Luria Beratni broth supplemented with sub-MIC concentration of drug meropenem in combination with inhibitors tazobactam, ZINC01807204 and ZINC02318494 (at fixed concentration of 4 µg/ml) in confocal dishes [13]. The control was taken without any drug. The media was inoculated and incubated at 37^ο^C for 4 hours. The media was removed and dishes were gently washed with MOPS (pH 7.0) to wash off extra media. Cells were then stained with propidium iodide (PI) and SYTO 9 (1∶1) for one hour. The fluorescence emission was observed using confocal scanning laser microscope FluoView FV1000 (Olympus, Tokyo, Japan). The optimal excitation wavelengths were 480 nm for PI and 545 nm for SYTO 9 and the optimal emission wavelengths were 500 and 610 nm, respectively. The images were obtained from atleast three randomly picked positions. The images of the control and in presence of known as well as novel inhibitors were averaged and compared.

### Cytotoxicity and MTT Assay

Cytotoxic effects of ZINC01807204 and ZINC02318494 against HeLa cell lines was determined by a rapid colorimetric assay, using MTT [14]. In this assay soluble MTT is metabolized by mitochondrial enzyme activity of viable tumor cells, into an insoluble colored formazan product. Subsequently formazan was dissolved in DMSO and measured spectrophotometrically at 565 nm. Briefly, human cervical cancer cell line HeLa was grown in DMEM medium supplemented with 10% heat inactivated fatal bovine serum at 37°C and 5% CO_2_ in a humified incubator. For assay 200 µl of cell suspension (approx. 5* 10^3^ cells) were seeded in 96 well plate. Cells were treated with five different concentrations (concentrations above and below 4 µg/ml) of both inhibitors (ZINC01807204 and ZINC02318494) (1 µg/ml, 2 µg/ml, 3 µg/ml, 4 µg/ml and 5 µg/ml) for 3 different time points viz. 24 hours, 36 hours and 48 hours. Followed by treatment, media containing inhibitors was replaced by fresh media containing 20 µg/ml MTT and was again incubated at 37°C for 3 hours. The formazan crystals formed were dissolved by adding 100 µl DMSO with constant shaking at 37°C. Absorbance was then determined at 565 nm by enzyme-linked immunosorbent assay (ELISA) plate reader. Each inhibitor concentration was assayed in 3 wells and repeated 3-times. The standard deviation was calculated by standard methodology and significance was calculated by student t-test.

### Determination of IC_50_ and Inhibition Constant (K_i_)

IC_50_ was determined by direct competition between beta-lactamase substrate nitrocefin and inhibitors under properly controlled experiments. Various concentrations of clavulanic acid, tazobactam, sulbactam, ZINC01807204 and ZINC02318494 were pre-incubated with 1 nM purified KPC-2 in 50 mM sodium phosphate buffer pH 7.0 for 5 minutes at 30°C before addition of substrate nitrocefin [15], [16]. The rate of hydrolysis of nitrocefin was monitored by the change in absorbance due to cleavage of β-lactam ring at 482 nm using Shimadzu UV-VIS Spectrophotometer UV-1800. The IC_50_ values were obtained by plotting percent residual enzyme activity on nitrocefin versus log of inhibitor concentration using OriginPro 8. The 50% inhibitory concentration (IC_50_) was defined as the concentration of the inhibitor that inhibited hydrolytic activity of the enzyme by 50%.

The inhibition constant, K_i_, was calculated from the IC_50_ value by applying the Cheng-Prusoff correction [17]



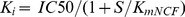
(1)


Here S and K_m_ corresponds to concentration and Michaelis-Menten constant of nitrocefin respectively. Kinetic parameters K_m_ and k_cat_ for nitrocefin were obtained in a separate experiment by nonlinear least-squares fitting of the data using Michaelis-Menten equation.

## Results

### Susceptibility testing of BL21 (pQE-2-KPC-2) cells

The effect of carbapenems alone and in combination with various inhibitors on *E. coli* cells transformed with *bla*_KPC-2_ is summarised in Table 1. The cells were found to be resistant to all carbapenems used with MICs of 64 µg/ml, 64 µg/ml and 128 µg/ml for imipenem, meropenem and ertapenem respectively. The commercial β-lactam-β-lactamase inhibitors inhibitors failed to show any significant activity and their MICs in combination with carbapenems were also high. In contrast to this, lowest MICs of carbapenems were obtained in combination with ZINC01807204 and ZINC02318494. Meropenem and ertapenem depicted best results with eight fold reduction in MIC while for imipenem MIC was reduced by four fold in combination with screened inhibitors. Our data revealed that MIC value was lowered from 64 µg/ml to 8 µg/ml and 128 µg/ml to 16 µg/ml for meropenem and ertapenem respectively and from 64 µg/ml to 16 µg/ml.

**Table 1:** MICs of carbapenems alone and in combination with inhibitors for *E. coli* BL21 transformed with recombinant *bla*_KPC-2_ from *Klebsiella pneumoniae*.

Antimicrobial Agents	MIC (µg/ml)	
	BL21 (pQE2-KPC-2)	BL21 (Null plasmid)
Imipenem	≥64	0.25
Imipenem+tazobactam	32	0.25
Imipenem+sulbactam Imipenem+clavulanic acid	64 32	0.25 0.125
Imipenem+ZINC01807204	16	0.125
Imipenem+ZINC02318494	16	0.25
Meropenem	≥64	0.125
Meropenem+tazobactam	16	0.0625
Meropenem+sulbactam Meropenem+clavulanic acid	32 16	0.125 0.625
Meropenem+ZINC01807204	8	0.125
Meropenem+ZINC02318494	8	0.0625
Ertapenem	≥128	0.25
Ertapenem+tazobactam	32	0.25
Ertapenem+sulbactam Ertapenem+clavulanic acid	64 32	0.125 0.25
Ertapenem+ZINC01807204	16	0.125
Ertapenem+ ZINC02318494	16	0.25

Clavulanic acid, tazobactam, sulabactam, ZINC01807204 and ZINC02318494 were used at fixed concentration of 4 µg/ml. All experiments were repeated atleast thrice.

### Live/Dead Cell Analysis

**Figure 2:**
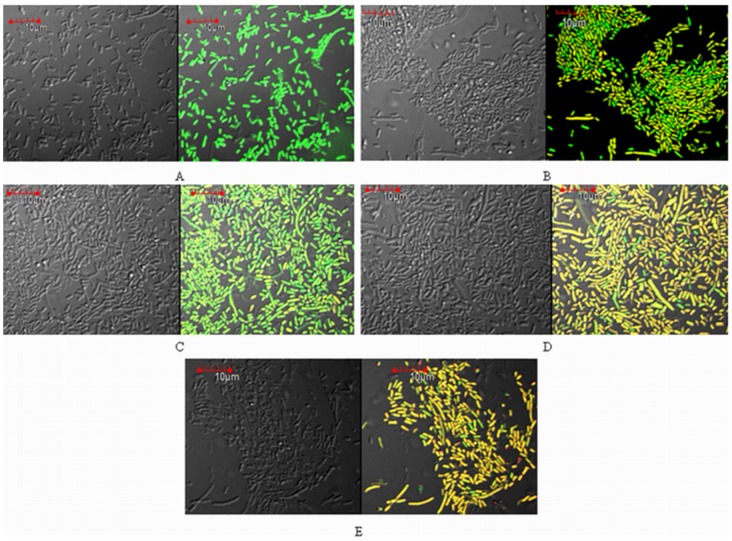
CLSM images of *E. coli* BL21 transformants harbouring *bla*_KPC-2_ 4 hours after treatment with sub-MIC concentration of (B) Meropenem (C) Meropenem + tazobactam (D) Meropenem + ZINC01807204 (E) Meropenem + ZIC02318494 (A) Control, no treatment.

To check the efficacy of screened non β-lactam inhibitors against commercial inhibitors, viability of cloned *E.coli* BL21 cells was analysed in presence of these inhibitors using confocal laser scanning microscopy (Figure 2). In absence of any antibiotic or inhibitor (control) cells appeared with undamaged and healthy cell membranes (cells were stained green). Whereas bacterial cells treated with sub-MIC concentration of meropenem, in combination with inhibitors (ZINC01807204 and ZINC02318494), showed extensive damage to their cell membranes as shown in the images (Figure 2D & 2E). Apparently 90–95% cells illustrated binding of PI and Syto 9 and hence displayed an intermediate colour in the image (yellowish orange), indicating that cells are affected. In contrast to this, treatment with sub-MIC concentration of meropenem alone and in combination with tazobactam affected the viability of bacterial cells to much lesser extent. A big proportion of cells (60–70%) cells appeared green with normal cell membranes and only small proportion of cells seemed affected (stained yellow) (Figure 2B & 2C).

### Toxicity of screened inhibitors

**Figure 3:**
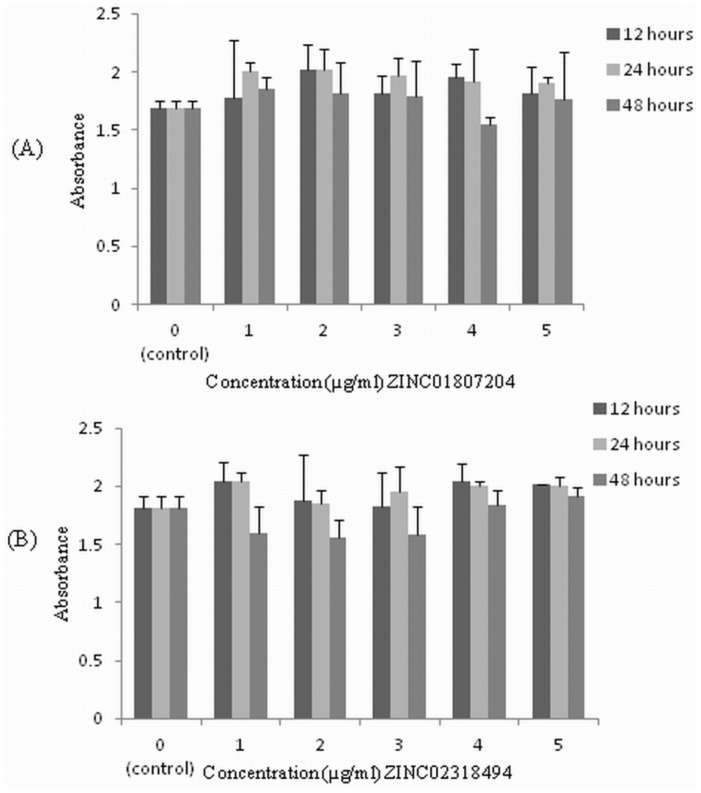
Effect of different concentrations of screened inhibitors on HeLa cell line for three different time durations (12 hours, 24 hours & 48 hours) determined by MTT assay. Percent cell survival in the control group was assumed 100%. (A) ZINC01807204 (B) ZIC02318494. n = 3, p<0.05.

The in-vitro cytotoxicity assay of novel inhibitors was carried out on human cervical cancer cell line (HeLa) at five different concentrations and for three different incubation time periods (Figure 3). The inhibitors were found to be non-toxic and did not show any significant cell lysis even after 48 hours of incubation and at concentrations more than two folds higher than their MIC value.

### Inhibition assays on purified KPC-2

**Figure 4:**
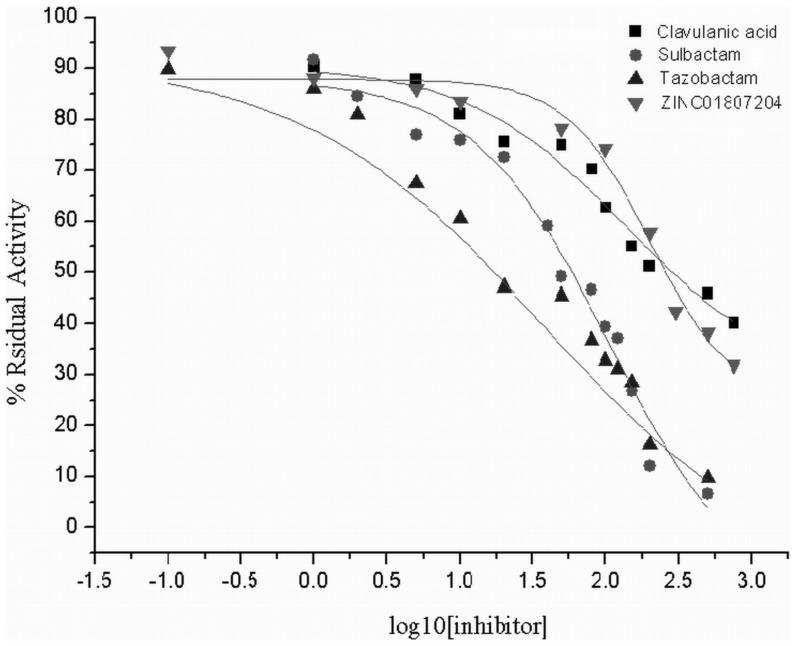
Determination of IC_50_ values for different inhibitors. The residual activity of KPC-2 after 5 minutes pre-incubation with varying concentrations of different inhibitors as monitored by the hydrolysis of 100 mM nitrocefin.

Studies assessing the half maximal inhibitory concentration IC_50_ values for ZINC01807204 have been measured and compared with Clavulanic acid, tazobactam and sulbactam for KPC-2. The results are presented in Table 2 and Figure 4. None of the inhibitors tested were very strong in inhibiting KPC-2 enzyme activity. Among all five inhibitors tested, tazobactam showed best inhibitory potency with lowest IC_50_ value of 98.79 µM followed by sulbactam at 106.09 µM. Clavulanic acid exhibited IC_50_ value of 136.93 µM. The non-β-lactam inhibitor ZINC01807204 displayed highest IC_50_ value of 200.29 µM.

**Table 2:** Half maximal inhibitory concentration values for ZINC01807204 and other β-lactamase inhibitors determined after 5 minute of incubation with KPC-2.

Inhibitor	IC_50_ (µM)	K_i_ (µM)
Clavulanic acid	136.93	29.96
Sulbactam	106.09	23.21
Tazobactam	98.79	21.61
ZINC01807204	200.29	43.82
ZINC02318494	ND	ND

*IC_50_* Half maximal inhibitory concentration. Shown as arithematic mean (three repetitions on three separate days).

To establish the kinetic parameters of inactivation, nitrocefin was used as a reporter substrate. The K_m_ and k_cat_ values for KPC-2 were determined and found as 28±6 µM and 36±0.002 s^−1^, respectively (Figure S2). Among the inhibitors the affinity for enzyme KPC-2 was found highest for Tazobactam with K_i_ value of 21.61 µM closely followed by sulbactam and clavulanic acid with K_i_ value of 23.21 µM and 29.96 µM respectively. Novel inhibitor ZINC01807204 displayed lowest affinity for KPC-2 among all four at 43.82 µM. The IC_50_ and K_i_ value of ZINC02318494 cannot be determined due to its poor solubility at higher concentrations.

## Discussion

KPC type β-lactamases have limited the treatment options for Gram negative bacterial infections worldwide. KPC-2 type β-lactamase has spread from *K. pneumoniae* to many other *Enterobacteriaceae* at an alarming rate [3]. Furthermore existing β-lactamase inhibitors (clavulanic acid, sulbactam and tazobactam) have limited efficacy against KPC enzymes because they can easily hydrolyze these inhibitors. This explains for the high MICs obtained in case of β-lactam-β-lactamase inhibitor combinations [18]. Thus developing new and potential inhibitors is the urgent need of the hour to maintain the effectiveness of present day antibiotics and prevent this serious medical problem.

In our previous work, we selected two potential non-β-lactam compounds (ZINC01807204 and ZINC02318494) by structure-based virtual screening and docking against KPC-2 enzyme. These two novel inhibitors exhibited impressive Gold score and *X* score (binding energies) which were found better than sulbactam and tazobactam. In the present study the inhibitors ZINC01807204 and ZINC02318494 were tested specifically against carbapenems and characterized by MICs, CLSM and IC_50_ studies. The MIC analysis of *K. pneumoniae* clinical strain NP6 showed that it is highly resistant to carbapenems (Table S1). The commercial inhibitors as well as novel inhibitors were not able to rescue their activity as none of the inhibitor was able to reduce MIC to susceptible range. This can be explained because a number of other factors like outer membrane proteins play an important role in development of resistance phenotype. Since the novel inhibitors were designed in particular against KPC-2, we tested the efficacy of these two non-β-lactam inhibitors for their inhibition potency against carbapenems on transformed BL21 cells expressing recombinant KPC-2. In consistent with our previous findings, MIC analysis showed that transformed BL21 cells displayed the lowest MIC for carbapenem antibiotics in combination with ZINC01807204 and ZINC02318494. Meropenem and ertapenem displayed the best bactericidal activity along with screened inhibitors. The resistance of KPC-2 β-lactamase for β-lactam β-lactamase inhibitors was illustrated by high MICs obtained for commercial inhibitor combinations. Moreover the screened inhibitors were best in restoring the bactericidal activity of the carbapenems. The CLSM (Live/Dead cells) analysis also exhibited the efficacy of screened inhibitors in combination with meropenem. The CLSM images also clearly depicted the KPC-2 resistance against conventional β-lactamase inhibitors and susceptibility towards novel inhibitors. The screened inhibitors demonstrated better restoration of antibacterial activity against KPC-2 expressing BL21 cells when compared to conventional inhibitors. The cytotoxicity assay was carried out on human cancer HeLa cell line and the two inhibitors were confirmed as non-toxic at concentration much above their MIC values. The inhibition analysis on purified enzyme KPC-2 showed that tazobactam had the lowest IC_50_ value and the highest affinity for enzyme while ZINC01807204 had the lowest affinity for KPC-2. The K_i_ values for ZINC01807204 were close to that of clavulanic acid. In general increased/decreased inhibitor potency against the enzyme should directly correlate with *in vitro* growth inhibition. However in the present study the screened inhibitor (ZINC01807204) illustrated better activity in *in vitro* on bacterial cells but lesser affinity to purified KPC-2 enzyme in comparison with commercial β-lactamase inhibitors. ZINC01807204 does not possess a β-lactam ring (non-β lactam), hence it is not readily recognized by the enzyme, and perhaps that is the reason it displayed high IC_50_ and low affinity for KPC-2. The commercial β-lactam inhibitors have high affinity for enzyme KPC-2 owing to their β-lactam ring but at the same time they get easily hydrolyzed. Moreover it is not always necessary for an inhibitor to show activity against bacterial cells which is consistent to its activity on purified enzymes [9].

## Conclusion

Our study concludes that ZINC01807204 is a novel non-β-lactam inhibitor that competes for the active site of the KPC-2 and interacts non-covalently with key residues involved in β-lactam recognition and hydrolysis. Moreover being non β-lactam, it is not readily hydrolyzed by KPC-2, as was evident by the results. Hence, ZINC01807204 may be proposed as a suitable lead molecule for development of future drug candidates.

## Supporting Information

Figure S1 **Purification of KPC-2. SDS-PAGE of induced KPC-2 protein in supernatant and pellet of bacterial cells after sonication in lane 1 and 2 respectively.** Lane 3, 4 and 5 represents purified KPC-2 protein in subsequent elutions. The single band represents molecular mass of approximately 29 kDa. Lane M is protein marker.
(TIF)

Figure S2 **Michaelis-Menten curve of nitrocefin at 485 nm.**
(TIF)

Table S1 **MICs of carbapenems alone and in combination with inhibitors for carbapenem resistant clinical strain NP6 and susceptible MTCC strain of** ***Klebsiella pneumoniae*** **.**
(DOCX)
