# Supplementary figures and images for: Evaluation of Inhibitory Action of Novel Non β-Lactam Inhibitor against Klebsiella pneumoniae Carbapenemase (KPC-2)
Source: PLoS One. 2014 Sep 29;9(9):e108246. doi: 10.1371/journal.pone.0108246 (PMC4180753; doi:10.1371/journal.pone.0108246)

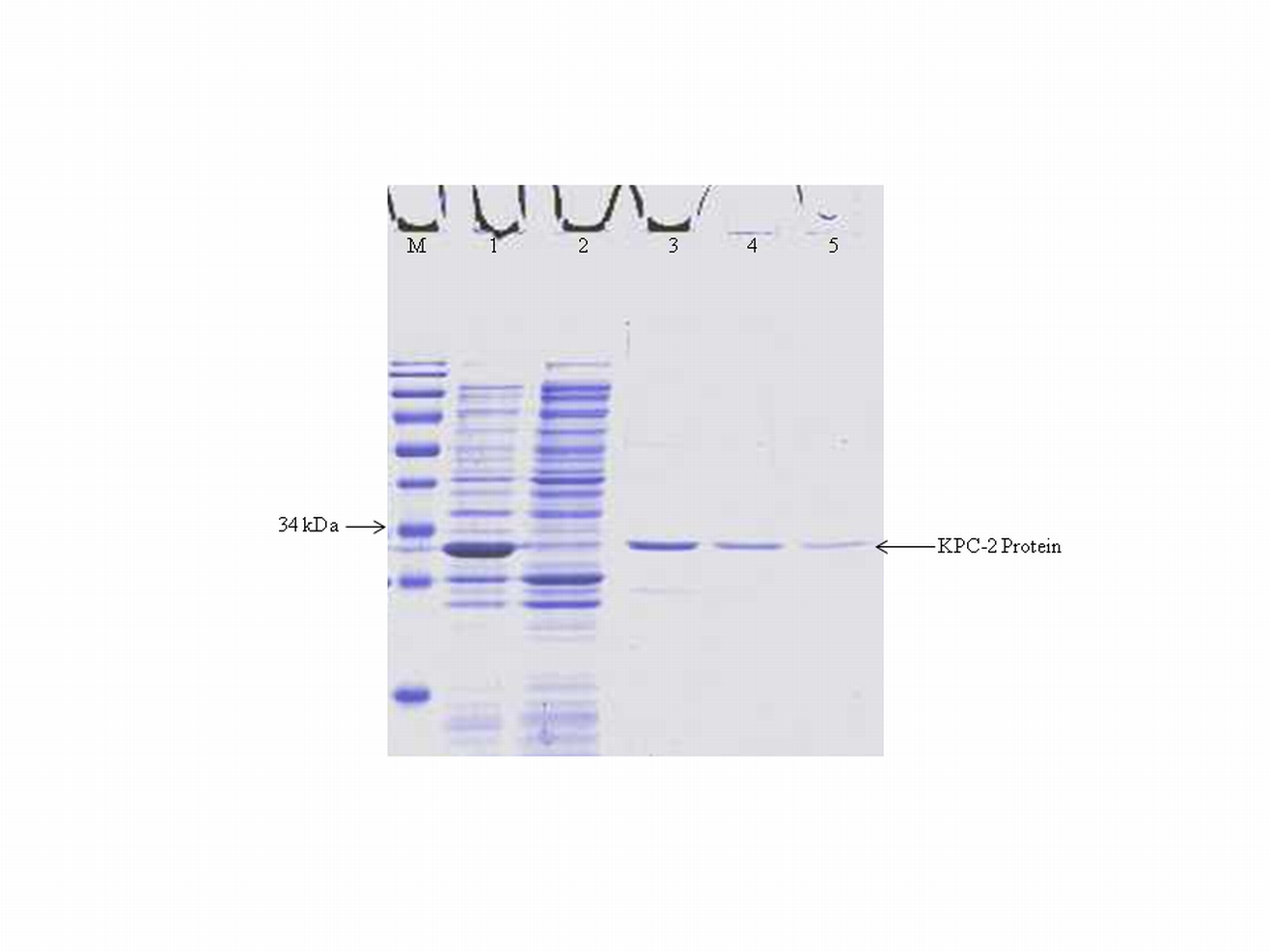

Supplement: Figure S1 — Purification of KPC-2. SDS-PAGE of induced KPC-2 protein in supernatant and pellet of bacterial cells after sonication in lane 1 and 2 respectively. Lane 3, 4 and 5 represents purified KPC-2 protein in subsequent elutions. The single band represents molecular mass of approximately 29 kDa. Lane M is protein marker. (TIF) [file pone.0108246.s001.tif]

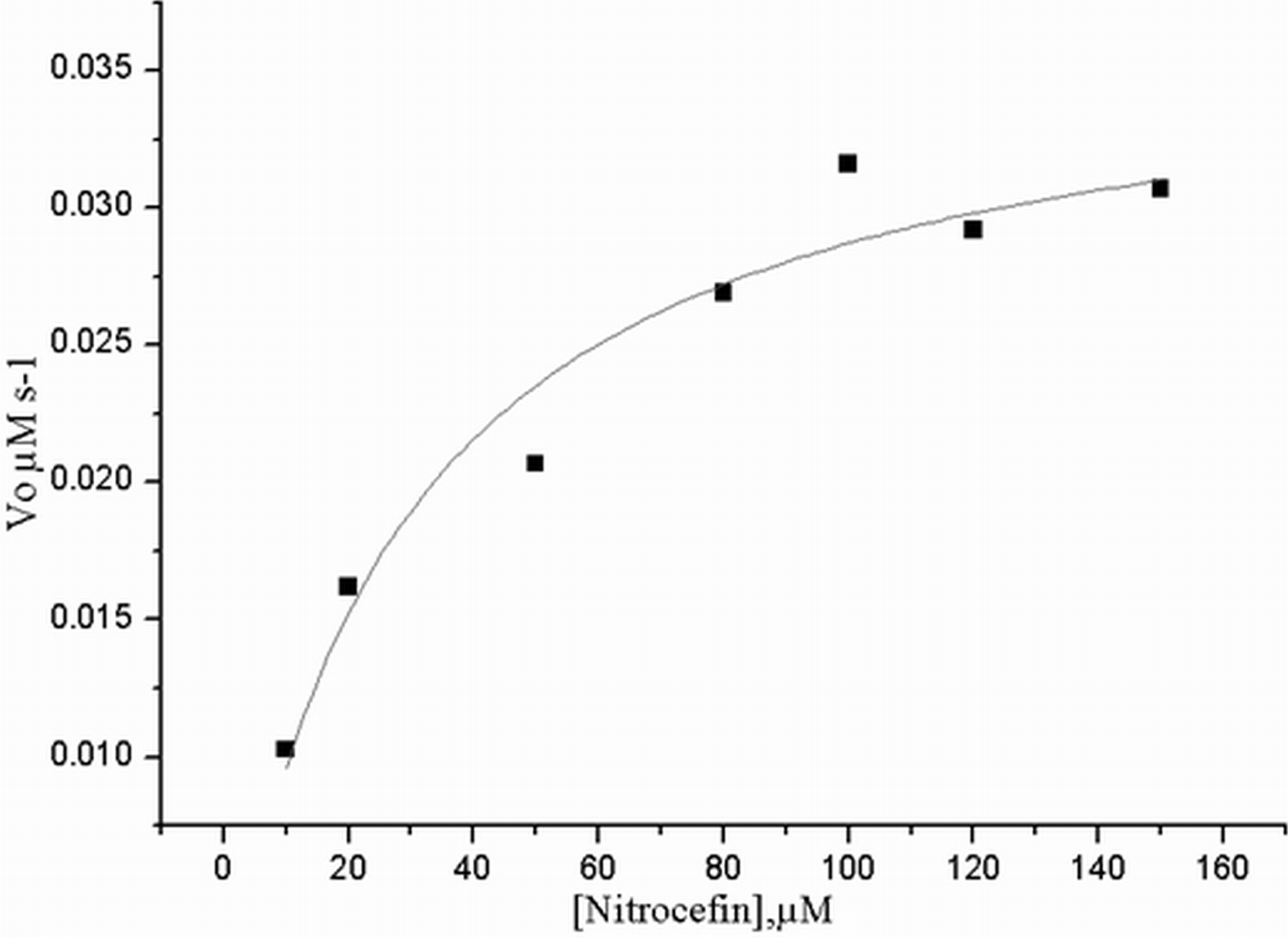

Supplement: Figure S2 — Michaelis-Menten curve of nitrocefin at 485 nm. (TIF) [file pone.0108246.s002.tif]
