# Supplementary material for: Evaluation of Inhibitory Action of Novel Non β-Lactam Inhibitor against Klebsiella pneumoniae Carbapenemase (KPC-2)
Source: PLoS One. 2014 Sep 29;9(9):e108246. doi: 10.1371/journal.pone.0108246 (PMC4180753; doi:10.1371/journal.pone.0108246)
Supplement: Table S1 — MICs of carbapenems alone and in combination with inhibitors for carbapenem resistant clinical strain NP6 and susceptible MTCC strain of Klebsiella pneumoniae . (DOCX) [file pone.0108246.s003.docx]

**Table S1**: MICs of carbapenems alone and in combination with inhibitors for carbapenem resistant clinical strain NP6 and susceptible MTCC strain of *Klebsiella pneumoniae*.

|  | **MIC (µg/ml)** |  |
| --- | --- | --- |
| **Antimicrobial agents** | **NP6 (Clinical strain)** | **MTCC 432** |
| Imipenem | ≥256 | 0.0625 |
| Imipenem+tazobactam | 256 | 0.0625 |
| Imipenem+sulbactam | 256 | 0.125 |
| Imipenem+clavulanic acid | 256 | 0.0625 |
| Imipenem+ZINC01807204 | 256 | 0.125 |
| Imipenem+ZINC02318494 | 256 | 0.0625 |
| Meropenem | ≥256 | 0.125 |
| Meropenem+tazobactam | 256 | 0.03125 |
| Meropenem+sulbactam | 256 | 0.0625 |
| Meropenem+clavulanic acid | 256 | 0.0625 |
| Meropenem+ZINC01807204 | 128 | 0.125 |
| Meropenem+ZINC02318494 | 128 | 0.125 |
| Ertapenem | ≥256 | 0.0625 |
| Ertapenem+tazobactam | 256 | 0.03125 |
| Ertapenem+sulbactam | 256 | 0.0625 |
| Ertapenem+clavulanic acid | 256 | 0.125 |
| Ertapenem+ZINC01807204 | 128 | 0.125 |
| Ertapenem+ ZINC02318494 | 256 | 0.0625 |

Inhibitors used at a fixed concentration of 4µg/ml
